# Use of Dexmedetomidine During an Emergent Exploratory Laparotomy in a High-Risk Cardiac Patient With an Intra-aortic Balloon Pump

**DOI:** 10.7759/cureus.40795

**Published:** 2023-06-22

**Authors:** Patrick Hussey, Charlotte A Snook, Hanna Hussey, Benjamin Tuck, Promil Kukreja

**Affiliations:** 1 Anesthesiology and Perioperative Medicine, University of Alabama at Birmingham, Birmingham, USA; 2 Anesthesiology and Perioperative Medicine, University of Alabama at Birmingham School of Medicine, Birmingham, USA

**Keywords:** dexmedetomidine, high risk cardiac, intra-aortic balloon pump, bispectral index, minimum alveolar concentration, sevoflurane

## Abstract

An intra-aortic balloon pump (IABP) may be placed preoperatively for high-risk patients with reduced ejection fraction or multivessel coronary disease undergoing non-cardiac surgery. Dexmedetomidine (DEX) has both anesthetic and cardioprotective effects, and little evidence is present on its effect on minimum alveolar concentration (MAC) and bispectral index (BIS). We present the case of a high-risk cardiac patient who was admitted and required fluid optimization prior to coronary artery bypass grafting (CABG). An IABP was placed after failure to tolerate intermittent hemodialysis (iHD). Bowel ischemia complicated this patient's course, necessitating an urgent exploratory laparotomy with the IABP in place. DEX and 0.3-MAC sevoflurane were successfully used without perioperative cardiac complications. Continuous BIS monitoring was performed to maintain an adequate level of anesthesia. DEX should be considered as an alternative anesthetic adjuvant in high-risk and medically complex patients.

## Introduction

Patients with extensive cardiac diseases may have an intra-aortic balloon pump (IABP) placed to augment cardiac output in a failing heart. Often, these patients may require emergent non-cardiac surgery with the IABP in place, which can complicate anesthetic management. High-risk cardiac patients often experience higher rates of cardiac mortality and morbidity perioperatively while undergoing non-cardiac surgery [1].

Preoperative placement of an IABP may reduce cardiac complications in these high-risk cardiac patients undergoing non-cardiac surgery [1]. The nature of an IABP, with augmentation of coronary perfusion pressure via increased diastolic aortic pressure and afterload reduction, decreases the cardiac workload and allows for less pharmacological myocardial support [2]. As many anesthetic agents decrease myocardial function, preload, and coronary perfusion pressure, an IABP increases the ability to titrate anesthetic agents.

Dexmedetomidine (DEX), a highly selective alpha-2 agonist, is an anesthetic agent that is often used as an infusion to produce hypnosis, anxiolysis, analgesia, and sedation. DEX does not have direct effects on cardiovascular function but does have significant indirect cardiovascular effects mediated by presynaptic alpha-2 adrenoreceptors in the locus coeruleus. This results in the inhibition of norepinephrine neuron activity, suppressing CNS sympathetic action, and decreasing catecholamine release [3,4]. This case investigates the use of DEX infusion and bispectral index (BIS) titration in high-risk cardiac patients supported by an IABP undergoing emergent non-cardiac surgery. Written informed consent was obtained from the patient to publish this case report.

## Case presentation

A 73-year-old, 115-kg man presented with a past medical history of heart failure with preserved ejection fraction (HFpEF), atrial fibrillation, deep vein thrombosis on warfarin, severe multivessel coronary artery disease status post-percutaneous coronary intervention, aortic valve replacement and mitral valve repair in 2002, and end-stage renal disease (ESRD) on intermittent hemodialysis (iHD). He was previously admitted for coronary artery bypass grafting (CABG) evaluation and discharged home with a plan for fluid optimization and CABG in two to three weeks. He was readmitted with symptomatic acute chronic heart failure.

The patient could not tolerate hemodialysis, and continuous renal replacement therapy (CRRT) was initiated with the placement of an IABP. A transthoracic echocardiogram (Video 1) was completed during hospitalization, showing a left ventricular ejection fraction of 35-40%, severe mitral regurgitation, severe tricuspid regurgitation, mild-moderately reduced right ventricular systolic function, and a well-seated prosthetic aortic valve with a normal gradient.

**Video 1:** Transthoracic echocardiogram during hospitalization Transthoracic echocardiogram in the parasternal long axis view demonstrating reduced left ventricular ejection fraction, reduced motion of the mitral valve, and a well-seated prosthetic aortic valve

**Figure 1:**
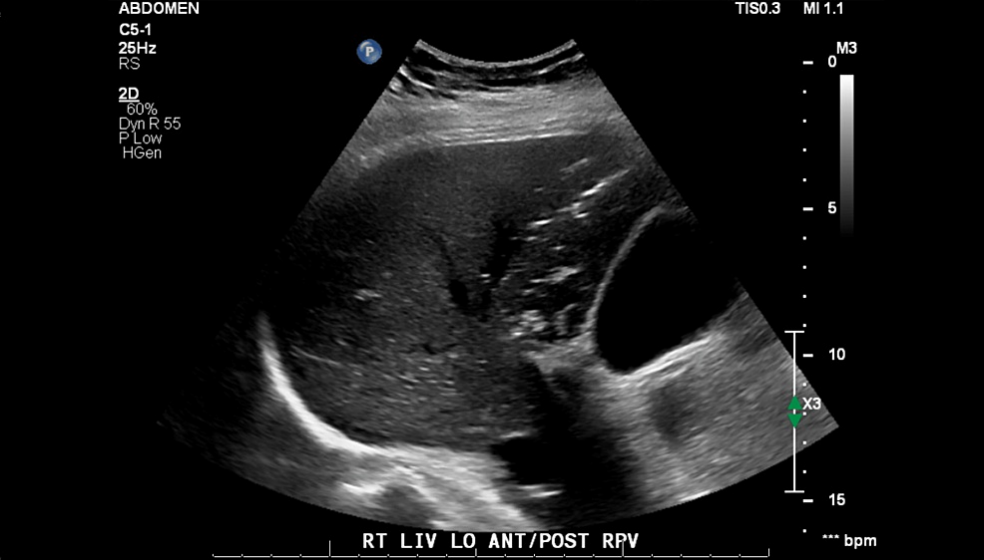
Liver ultrasound Liver ultrasound demonstrating air in the portal vein of the right lobe

**Figure 2:**
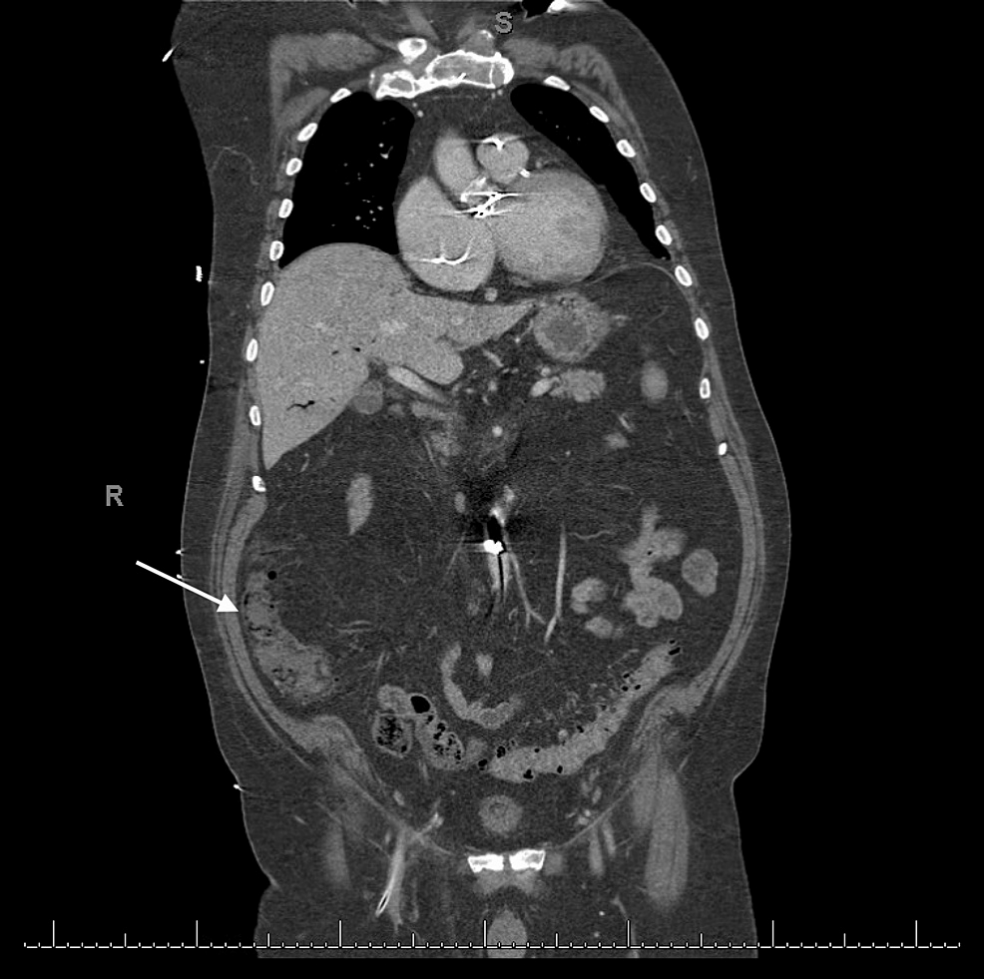
Computed tomography of chest and abdomen Computed tomography of chest and abdomen with air around the ascending colon (arrow)

His hospital course was complicated by severe abdominal pain in the intensive care unit (ICU), an increasing vasopressor requirement, and leukocytosis. Ultrasound imaging (Figure 1) revealed air in the portal vein, and computed tomography (Figure 2) demonstrated ascending colon pneumatosis. The IABP was noted to be in an appropriate position at this time. Laboratory values can be seen in Table 1.

**Table 1:** Patient laboratory results Table of laboratory results listing laboratory values prior to the operating room course, the last laboratory values prior to leaving the operating room, and the institutional normal reference range of values

	Prior to OR	End of OR	Normal level
pH	7.39	7.36	7.35–7.45
PaCO_2_	37	36	35–45 mmHg
PaO_2_	92	346	80–100 mmHg
BE	−2.3	−4.4	NA
Lactate	1.1	0.9	0.5–2.2 mMol/L
White blood cell	14.82		4–11 × 10^3^/cmm
Hemoglobin	10.5	10	13.5–17 g/dL
Hematocrit	32	31	39–50%
Platelet	207		150–400 × 10^3^/cmm
Sodium	136		133–145 mMol/L
Potassium	4.3		3.1–5.1 mMol/L
Chloride	96		97–108 mMol/L
Bicarbonate	21		22–32 mMol/L
BUN	19		5–22 mg/dL
Creatinine	1.9		0.7–1.3 mg/dL
Ionized calcium	1.28	1.27	1.12–1.32 mMol/L
Glucose	136	169	79–100 mg/dL

The patient arrived to the operating room for a proposed emergent exploratory laparotomy, right hemicolectomy, and small bowel resection. He presented with the IABP on 1:1 full augmentation, a right brachial arterial line, a right internal jugular central line with a pulmonary artery catheter, and a left internal jugular dialysis central line. He was receiving a vasopressin infusion at 0.03 units/min and a heparin infusion at 14 units/kilogram/hour. On arrival, the patient was tachycardic to 120 beats per minute with mean arterial pressures (MAP) of 70-80 mmHg and mean pulmonary pressures of 25-30 mmHg. Cardiac output was not measured during the case. Anesthesia was induced with 4 mg midazolam, 200 mcg of fentanyl, and 100 mg rocuronium to facilitate intubation. Maintenance sevoflurane was started at 0.6 age-adjusted minimum alveolar concentration (MAC) for surgical anesthesia. To support hemodynamics, given the patient's known reduced cardiac function, an epinephrine infusion at 0.05mcg/kg/min was initiated and increased to 0.08/mcg/kg/min, and vasopressin was increased to 0.05 units/min.

Given increasing vasopressor support, a DEX infusion started at 0.5 mcg/kg/min. A BIS monitor was placed after DEX initiation, with readings showing 20-30. Sevoflurane was reduced to 0.3 MAC with continued muscle paralysis. BIS of 30-40 was targeted given volatile concentration below MAC aware. Vasopressin and epinephrine were able to be reduced to 0.03 units/min and 0.05mcg/kg/min, respectively. Laboratory values prior to leaving the operating room can be seen in Table 1 above. The remainder of the case proceeded uneventfully, and the patient was transported back to the ICU intubated on the above infusions of vasopressin, epinephrine, and DEX with an open abdomen.

Postoperatively, the epinephrine was weaned off, and vasopressin remained at 0.03 units/min with DEX at 0.5 mcg/kg/min until postoperative day 2 for abdominal closure.

## Discussion

When caring for high-risk cardiac patients for non-cardiac surgery, anesthesiologists must consider the implications and management of mechanical circulatory support and the consequences of a volatile anesthetic. High-risk cardiac patients, despite IABP support, are at a higher risk for perioperative cardiac morbidity and mortality due to increased perioperative myocardial infarction and ventricular arrhythmias following non-cardiac surgical intervention [1]. Anesthetic management and optimization through invasive hemodynamic monitoring, medication management, and preoperative optimization may reduce mortality and morbidity; however, these patients still experience a high incidence of cardiac complications and hemodynamic instability [1]. Importantly, improper positioning of mechanical circulatory support devices can inadvertently cause complications such as retroperitoneal bleeding, stroke, dissection, and bowel ischemia. A chest and abdominal computed tomography scan of this patient confirmed proper positioning.

Given that this patient arrived at the OR with an IABP, tachycardic, and vasopressor infusion, we were concerned that a volatile anesthetic would cause hemodynamic instability. Therefore, the decision was made to infuse DEX and titrate sevoflurane based on BIS output to maintain an adequate level of anesthesia. Unfortunately, in this case, BIS values were acquired after sevoflurane and DEX initiation. With BIS and DEX utilization at the start of anesthetic management, the up-titration of hemodynamic support might have been avoided.

DEX produces its effect mainly by activating alpha-2 receptors in the central nervous system. Cardiovascular effects of DEX are sympatholytic and include a decrease in heart rate, systemic vascular resistance, MAP, and myocardial oxygen consumption. The reduction in heart rate prolongs diastolic perfusion and increases coronary perfusion pressure, decreasing the risk of myocardial ischemia in at-risk patients, provided cardiac output is not reliant on heart rate, as in a low cardiac output state [4]. Other desirable anesthetic effects of DEX include reducing the MAC [5]. It has been previously suggested that DEX may have myocardial protective effects in patients undergoing cardiac surgery and reduce postoperative cardiac mortality and the incidence of myocardial infarction in high-risk patients undergoing non-cardiac surgery [3,5]. Lab studies have demonstrated a reduction of myocardial inflammation, oxidative stress, and cellular apoptosis, though the exact mechanism is unknown [6].

There is decreased responsiveness to vasopressors in sepsis, but experimental data suggest that DEX may counteract this finding [7]. When comparing DEX to usual care group sedation practices in the ICU setting, DEX appeared to be associated with lower vasopressor requirements [8]. In this case, we noticed a more hemodynamically stable anesthetic with mechanical-assisted support, DEX infusion, and reduced MAC. Currently, the literature lacks support for this finding in the operating room setting, but it is reassuring that septic patients who received DEX in the ICU did not require more vasopressor support.

When deciding to reduce sevoflurane, we did not wish to do this at the expense of intraoperative awareness, which can have profound psychological disturbances [9-11]. While intraoperative awareness is rare, utilizing BIS may reduce this risk [12]. BIS (Medtronic, Minneapolis, MN, USA) uses an electroencephalogram (EEG) to generate a unitless number to calculate the hypnotic component of an anesthetic agent: 100 indicates an awake state, 0 is isoelectric, and 40-60 is an appropriate anesthetic depth [13]. The BIS value can vary with the type of anesthetic agent, electrocautery, neuromuscular blockers, temperature, and neurological conditions [12]. With the addition of DEX to a sevoflurane maintenance anesthetic targeting BIS levels 40-60, the mean sevoflurane end-tidal concentration was statistically significantly lower at 0.8% end-tidal concentration versus 1% end-tidal concentration. In addition, lumbar spine surgery patients receiving DEX had more hemodynamic stability, reduced intraoperative blood loss, and earlier extubation than patients who did not receive DEX [14]. DEX has also been shown to reduce BIS more than volatile anesthetics with more hemodynamic stability [15], which is especially important in a patient with an IABP.

## Conclusions

In the high-risk cardiac patient, supported by an IABP and on the verge of sepsis, DEX combined with BIS allows for reduced volatile anesthetic while maintaining the anesthetized state. In addition to cardioprotective effects at a cellular and physiological level, the clinical anesthesiologist’s addition of DEX and reduced volatile anesthetic allowed for greater hemodynamic stability in high-risk cardiac patients.
